# Case report: First case of undifferentiated embryonal sarcoma of the liver in a child with neurofibromatosis type 1, treated by hepatic chemoperfusion with transcatheter arterial chemoembolization

**DOI:** 10.3389/fonc.2022.981230

**Published:** 2022-10-24

**Authors:** Ksenia Sinichenkova, Ludmila Yasko, Dmitry Akhaladze, Anton Petrushin, Dmitry Konovalov, Ruslan Abasov, Yulia Mareeva, Olga Melekhina, Natalia Usman, Alexander Karachunsky, Galina Novichkova, Dmitry Litvinov, Alexander Druy

**Affiliations:** ^1^ Dmitry Rogachev National Medical Research Center of Pediatric Hematology, Oncology and Immunology, Moscow, Russia; ^2^ Anatoly S. Loginov Moscow Clinical Scientific Center, Moscow, Russia; ^3^ Research Institute of Medical Cell Technologies, Yekaterinburg, Russia

**Keywords:** undifferentiated embryonal sarcoma of the liver, malignant peripheral nerve sheath tumor, transarterial chemoembolization, chemoperfusion, neurofibromatosis type 1

## Abstract

**Introduction:**

In this report we firstly describe undifferentiated embryonal sarcoma of the liver (UESL) in a patient with neurofibromatosis type 1 (NF1), fatally complicated by synchronous malignant peripheral nerve sheath tumor (MPNST) with a highly aggressive metastatic course. The case also represents our first experience of chemoperfusion involving the transcatheter arterial chemoembolization (TACE) in a pediatric patient, applied as a treatment for UESL.

**Case presentation:**

A 13-year-old girl was diagnosed with NF1 and presented with a liver tumor identified as UESL by histological assessment. The tumor was refractive to the conventional first-line chemotherapy. The patient received hepatic chemoperfusion with TACE, which was efficacious; however, the overall curative outcome was unsatisfactory due to synchronous unresectable retroperitoneal MPNST with mesenteric metastases and ultimate progression of the UESL.

**Conclusion:**

This is the first reported case of UESL in a patient with NF1. The results demonstrate the efficacy of hepatic chemoperfusion with TACE in pediatric UESL.

## Introduction

Neurofibromatosis type 1 (NF1) is an autosomal-dominant disorder with complete penetrance, variable expressivity, and multisystem clinical manifestations. NF1 is caused by germline pathogenic mutations in the eponymous tumor suppressor gene *NF1* (17q11.2). Birth prevalence of pathogenic mutations in *NF1* is about 1 : 2500–3500. The patients most typically present with benign soft tissue skin nodules (neurofibromas) which may transform into malignant peripheral nerve sheath tumors (MPNST) (1). Apart from MPNST, NF1 complications include a spectrum of other malignant neoplasms. Overall, NF1 is justly regarded as a tumor predisposition syndrome.

The NF1-associated tumorigenesis conventionally involves biallelic inactivation of *NF1*, which encodes the neurofibromin protein involved in regulation of several cytoplasmic cascades, notably the RAS/RAF/MEK/ERK signaling pathway (1). MPNST, which predominantly arise within preexisting plexiform neurofibromas, are encountered in about 10% of patients with NF1 (2).

Apart from MPNST, sarcomas rarely occur in NF1, which is consistent with the lack of obvious pathogenetic relationship between other sarcomas and NF1. In this article, we describe undifferentiated embryonal sarcoma of the liver (UESL) in a pediatric patient with NF1. To the best of our knowledge, this is the first reported case of UESL in NF1. Noteworthy, the patient developed UESL synchronously with MPNST. The tumor responded to regional chemoperfusion with transcatheter arterial chemoembolization (TACE) indicated for unresectable liver cancers including UESL (3).

## Case presentation

**Figure 1:**
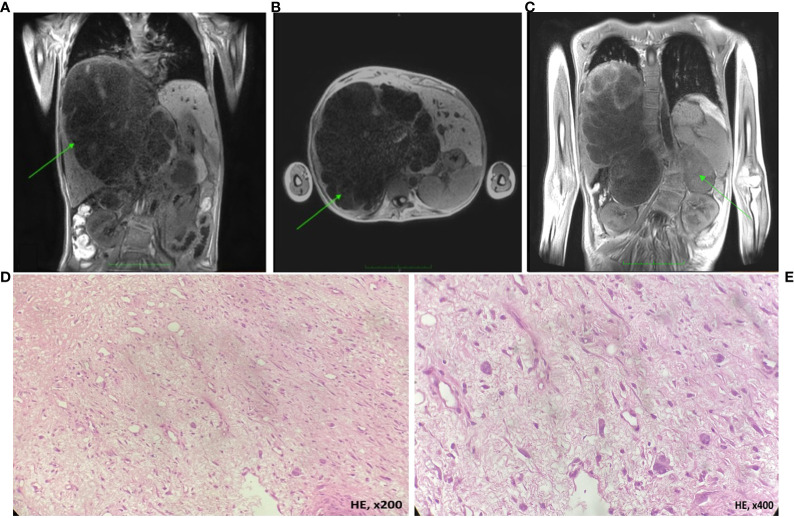
MRI of the abdominal cavity and morphological assessment of the liver tumor biopsy (UESL; hematoxylin-eosin, HE). **(A, B)** Images reveal a massive cystic formation in the right lobe of the liver, encompassing the segments S1, S5, S7, S8, and partially S4 and S6, measured 19 × 18.8 × 19.3 cm (3580 cm^3^), with the signs of periportal infiltration. The inferior vena cava at the level of tumor mass is compressed and pushed aside. The right and middle hepatic veins, as well as the arteries, pass through the mass. The portal vein at the level of the bifurcation and its right branch pass through the mass. Classified as PRETEXT stage III C1, E0, H0, F1, N0, M0, P2, V2; **(C)** a T2-weighted image shows a solitary tumor node, 8 × 6 × 8 cm. Microscopically the lesion was presented by short bundles and sheets of spindle-shaped or irregularly shaped cells and collagenous stroma with myxomatous areas **(D)**. Neoplastic cell exhibited moderate nuclear-cytoplasmatic ratio, nuclei varied in site and shape from ovoid to polygonad. The cytoplasm of tumor cell was eosinophilic or optically empty **(E)**.

A 13-year-old girl with severe scoliosis, café-au-lait macules, and soft skin nodules was clinically diagnosed with NF1. Next-generation sequencing (NGS) of DNA isolated from peripheral blood leukocytes of the patient revealed a previously undescribed heterozygous loss-of-function variant c.4771del, p.(Ser1591ValfsTer33) in the *NF1* gene (NM_001042492.2; hg 19). The patient’s parents had no clinical manifestations of neurofibromatosis; nevertheless, they also underwent genetic testing which confirmed *de novo* origin of the identified genetic variant. One year later, a scheduled ultrasound examination of the abdominal organs revealed 10 × 8 × 2 cm masses in the right lobe of the liver and in the subhepatic space retroperitoneally. Considering the established diagnosis of NF1, pheochromocytoma or paraganglioma were suspected. After few weeks of observation, the patient presented with abdominal pains and fever and was urgently hospitalized. Examination at admission revealed cachexia and enlarged liver without jaundice. Laboratory blood tests revealed no critical liver function abnormalities (serum albumin 33 g/L, ALT 35 U/L, AST 57 U/L, total bilirubin 8 µmol/L, direct bilirubin 3.7 µmol/L, GGT 433 U/L elevated, and LDH 541 U/L elevated to 3-fold normal), without significant increase in tumor markers (NSE 30 ng/mL, AFP 8.28 ng/mL, and β-hCG <1.2 mIU/mL). Magnetic resonance imaging (MRI) of the abdominal cavity (**Figures 1A, B**) revealed a massive tumor in the right lobe of the liver, about 3580 cm^3^ and rapidly growing (by 441% in 2 months). A solitary node observed in the projection of the left adrenal gland at the time of the diagnosis was regarded as a metastasis to lymph nodes (**Figure 1C**).

The patient underwent a laparoscopic liver tumor biopsy for histological assessment. The tumor tissue was represented by short bundles and sheets of spindle-shaped or irregularly shaped cells with a moderate nuclear-cytoplasmic ratio; the tumor cells had nuclei of varying size and shaped from ovoid to polygonal, the chromatin сlumpy or hyperchromic, the cytoplasm weakly eosinophilic or optically empty; the stroma presented with low collagen content and myxomatous areas (**Figures 1D, E**). Immunohistochemical tests revealed uniform expression of INI1 and Vimentin and focal expression of Glypican3, Desmin, trypsin, chymotrypsin, PCK AE1/AE3, and MDM2 by tumor cells and negative reaction with antibodies to CD10, CD68, SMA, Calponin, MSA, Myf4, PLAP, Sall4, Synaptophysin, HSA, S100, and CD34; the Ki67 proliferation index constituted 70%. The results of histological assessment produced the diagnosis of undifferentiated embryonal sarcoma of the liver (UESL).

With consideration to the huge volume of the tumor and the possible infiltration of the portal vein, the decision was made to commence preoperative chemotherapy according to the CWS-2009 protocol. A check-up MRI performed after three cycles of chemotherapy (ifosfamide, actinomycin D, and vincristine) revealed a 37% increase in tumor volume; at that, the solitary node in the abdominal cavity retained its size. Radical tumor resection is known to be a key survival factor in patients with UESL (4). However, in this case, a radical surgery, optionally with the use of transplantation technologies, was excluded due to the portal vein infiltration.

The choice of transarterial chemoembolization (TACE) and chemoperfusion (a.k.a. transarterial chemotherapy, TACT) was based on successful use of these approaches in adult patients with unresectable liver tumors, such as hepatocellular carcinoma and other hypervascular liver neoplasms including UESL (5–7).

**Figure 2:**
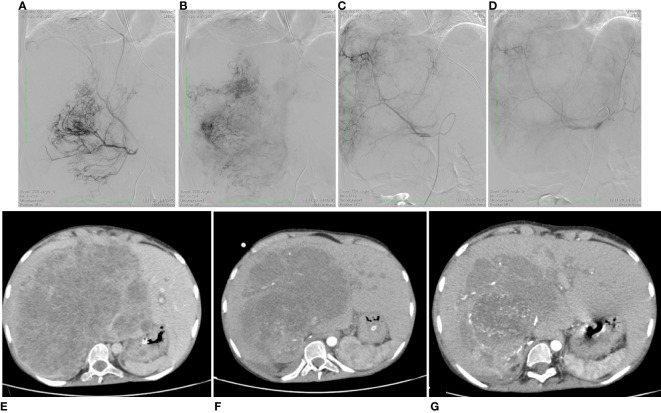
Angiographic imaging of the UESL-supplying vessels and MRI images showing favorable dynamics of the liver tumor volume. **(A)** Proximal portion of the right diaphragmatic artery supplying UESL; **(B)** distal portion of the right diaphragmatic artery supplying UESL; **(C)** hepatic artery proper before chemoembolization; **(D)** hepatic artery proper after chemoembolization, with the catheter left in place for chemoperfusion. **(E)** before the interventional procedures; **(F)** after the first session of chemoembolization; **(G)** after the second session of chemoembolization.

The method consists of 3 steps: 1-angiography; 2- embolization of the tumor-supplying blood vessels; and 3- chemoperfusion of the tumor. Primary angiography revealed that the liver tumor received its blood supply not only from the proper hepatic artery, but had developed its own vasculature of collateral arteries, extending from the branch of the right diaphragmatic artery, as well as from the superior mesenteric artery and the right renal artery. The patient underwent endovascular occlusion of the right diaphragmatic artery with 500 µm microspheres. The transcatheter oil chemoembolization applied a mixture of lipiodol and doxorubicin (50 mg) to fill the branches of the right diaphragmatic artery (**Figures 2A, B**) and the collateral arteries nourishing the tumor from the superior mesenteric artery. After that, 25 mg of doxorubicin over 2 hours and 100 mg of cisplatin over 5 hours were infused through a catheter placed in the right hepatic artery.

The chemotherapy infusion into the right hepatic artery was performed under sedation to exclude the patient’s movement and catheter displacement. Few days after the procedure the patient presented with abdominal pains, which required narcotic analgesia, and fever, corresponding to the development of post-embolization syndrome; these symptoms resolved in two weeks. The procedure resulted in tumor response [a 20% reduction in the tumor volume according to computed tomography; (**Figures 2E, F**)], which served an indication for the second session.

The second chemoembolization used microspheres 200–400 µm in diameter loaded with oxaliplatin (50 mg) infused in the collateral arteries stemming from a branch of the right diaphragmatic artery, the superior mesenteric artery, and the right renal artery nourishing the sarcoma, and lipiodol with doxorubicin (50 mg) infused in the hepatic artery proper (**Figures 2C, D**). Chemoperfusion involved doxorubicin at a dose of 25 mg for 2 hours and oxaliplatin at a dose of 25 mg for 1 hour through catheter inserted in the proper hepatic artery.

The second session of chemoembolization/chemoperfusion was accompanied by a more severe post-embolization syndrome, presenting with intense abdominal pains unresponsive to morphine. To alleviate the symptoms, the patient received epidural anesthesia of morphine and naropin with a positive effect. The pain syndrome persisted for 1.5 months. After the second session, the tumor decreased by another 55% to a volume of 2144 cm^3^, though the periportal infiltration persisted (**Figure 2G**); noteworthy, the solitary tumor node on the left, interpreted as a paraaortic lymph node metastasis, lacked significant dynamics. Considering the significant reduction in the liver tumor volume, the decision was made to attempt its resection. The surgical scope of the planned intervention included extended right-sided hemihepatectomy with removal of the retrohepatic portion of the inferior vena cava and its replacement with a synthetic prosthesis under conditions of total vascular isolation and cold perfusion of the liver, and implantation of the left hepatic vein into the prosthesis.

**Figure 3:**
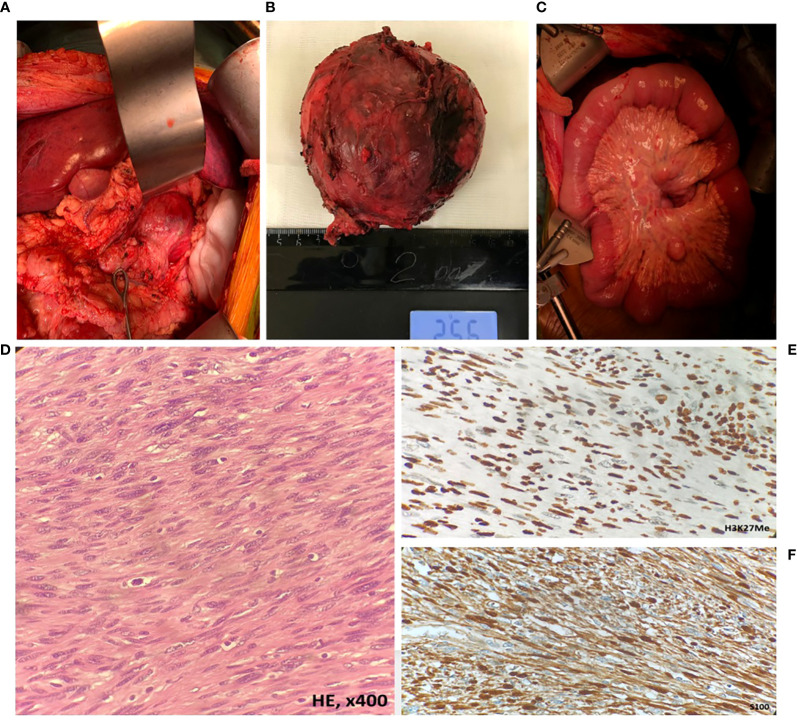
Intraoperative appearance and morphological assessment of the retroperitoneal tumor node and mesenteric tumor foci. **(A, B)** The solitary tumor node in the retroperitoneum (MPNST); **(C)** metastatic foci in the mesentery (MPNST); **(D)** MPNST spindle-shaped cells with scanty cytoplasm with hyperchromatic; **(E)** MPNST cells showed retained H3K27Me expression; **(F)** diffuse immune reactivity for S-100.

The patient was operated by “J” laparotomy; examination in the retroperitoneal space on the left revealed a tumor node 10 × 10 cm, hardly resembling a lymph node metastasis by gross appearance, removed surgically (**Figures 3A, B**). Intraoperative examination revealed multiple diffuse lesions in the mesentery of the small intestine, reaching 5.2 cm in diameter (**Figure 3C**); with regard to the baseline diagnosis of neurofibromatosis type 1, these lesions were preliminary identified as neurofibromas. Morphological examination of the distant tumor node and the mesentery foci revealed their histological identity: long intertwining bundles of medium-size spindle cells, with large ovoid nuclei with dispersed chromatin, embedded in the fibrous stroma. The tumor also contained cellular elements with shapeless hyperchromic anaplastic nuclei and zones of chondroid differentiation (**Figure 3D**). Immunohistochemical tests revealed partial loss of S100, H3K27me3, and SOX10 expression; subtotal loss of р16; and negative reaction with specific antibodies to SMA, Calponin, TRK, and Myogenin (**Figures 3E, F**). Thereby, morphological examination confirmed the presence of synchronous tumor process identified as malignant peripheral neural sheath tumor (MPNST) grade 2 with peritoneal metastasis.

Molecular examination of the UESL and MPNST tumor samples by NGS Qiaseq (Qiagen) for a custom panel of genes known to be involved in etiology and pathogenesis of solid tumors in children, supported the presence of the previously identified germline genetic variant *NF1* c.4771del, p.(Ser1591ValfsTer33). The multiplex ligation-dependent probe amplification assay (MLPA) revealed a heterozygous deletion of *NF1* coding sequence in MPNST tissue.

Thus, the final diagnosis accounted for the synchronous development of two tumors — UESL and MPNST of the retroperitoneal space with metastatic lesions in the mesentery. Given the unfeasibility of the radical resection for MPNST, traumatic resection of the liver tumor was abandoned. The patient was declared incurable and prescribed with palliative course of MEK inhibitor trametinib at 2 mg daily doses orally. Upon the commencement, the patient developed intractable diarrhea with a morphological substratum of intestinal ganglioneuromatosis, leading to reduced absorption of the drug. Trametinib treatment did not lead to the control of the UESL and the local progression was noted. However, primary and metastatic lesions of MPNST presented stable. The patient received trametinib for 1.5 months before dying of UESL progression.

## Discussion

Patients with NF1 have significantly increased risks of developing benign and malignant tumors of neurogenic and non-neurogenic origin (8–11). The spectrum is dominated by tumors of neurocutaneous origin, but includes a variety of other malignancies (**Table 1**).

**Table 1:** The incidence of malignant neoplasms in patients with NF1.

Tumor type	Incidence in NF1
Malignant peripheral nerve sheath tumor (MPNST)	8–15% (12)
Optic nerve glioma	25% (13)
Gastrointestinal stromal tumor	1.2–25% (14)
Melanoma	1% (12)
Pheochromocytoma	1–5.7% (12)
Juvenile myelomonocytic leukemia	less than 1% (12)
Embryonal rhabdomyosarcoma, acute lymphoblastic leukemia, neuroectodermal tumors, prostate adenocarcinoma, squamous cell lung cancer, undifferentiated pleomorphic sarcoma, osteosarcoma	less than 1% (12)

The lifelong risk of MPNST in patients with NF1 is estimated 8–13% (15). MPNST is the leading cause of death among patients with NF1 aged under 40, responsible for 60% of lethal cases in this group (9). Survival in children with MPNST is critically affected by metastatic character of the disease (33.3% compared with 82.8% in children presenting with localized disease) (10). Surgical treatment of MPNST is the only effective option for such patients; at that, 17–53% of pediatric MPNST are qualified as initially unresectable. The utility of chemotherapy in pediatric MPNST is limited: the majority of unresectable cases show moderate chemotherapy response, which tends to be lower in patients with NF1 (11, 15, 16). Reliable targeted therapy options are missing as well: so far, none of the clinical studies have succeeded to improve event-free survival in patients with MPNST using targeted therapy (17).

The current case provides a unique example of UESL developing in the clinical context of NF1. According to the literature, UESL typically harbor translocations t (11, 13) (q13.1;q13.42) involving *MALAT1* or t (2, 13)(q31.1;q13.42) involving *AK023515*, an uncharacterized noncoding gene; alternatively, an inv (13, 13)(q13.42;q13.43) inversion involving a Kruppel-type zinc-finger protein-encoding gene *PEG3* may be encountered (18, 19). These tumors may also harbor mutations in *TP53* including somatic deletions and missense substitutions (19). However, we found no published examples of sequence alterations involving *NF1*, which encodes a negative cytoplasmic regulator of the RAS/RAF/MEK/ERK signaling pathway, and no evidence of aberrant activation of this pathway in UESL, with a reservation that the clinical data are scarce due to the rarity of this pathology. It should be also emphasized that liver tumors are not characteristic of NF1. The most prevalent hepatic cancer of childhood, hepatoblastoma, has been associated with other hereditary tumor predispositions including familial adenomatous polyposis, Beckwith-Wiedemann, and Li-Fraumeni syndromes, as well as Edwards syndrome (trisomy 18) and glycogen storage disease type I, but not NF1 (20).

The frequency of certain malignant neoplasms among patients with NF1 significantly exceeds their population incidence rates; these tumors are conventionally known as NF1-associated and their spectrum is limited (**Table 1**). Noteworthy, such tumors are genetically distinct from similar tumors that occur sporadically.

**Table 1** shows that, apart from the ‘classical’ NF1-associated tumors characterized by biallelic inactivation of *NF1*, the spectrum includes certain diagnoses (e.g. rhabdomyosarcoma, osteosarcoma, and lung cancer) with higher incidence in NF1 compared with total population rates, but harboring no biallelic inactivation of NF1 (similarly with the current case). Although the loss-of-function mutation in a single allele of *NF1* apparently contributes to the oncogenesis, such tumors as UESL cannot be considered NF1-associated.

*NF1* is a universal tumor suppressor gene, dysfunction of which may contribute to RAS/RAF/MEK/ERK hyperactivation *via* haploinsufficiency. For certain conditions, e.g. neuroblastoma, *NF1* mutations have been associated with adverse prognosis (12). In order for a particular cell to become cancerous, both alleles of a *NF1* gene must be mutated, according to the ‘two-hit’ concept, and the majority of NF1-associated tumors exhibit biallelic inactivation of *NF1* (12– 14). Over 80% of germline mutations in *NF1* are inactivating, predicted to result in almost complete exclusion of the affected allele from transcription and/or protein production (22). The biallelic inactivation of *NF1*, revealed by us in the synchronous MPNST, is characteristic of NF1-associated tumors (23). And reciprocally, the lack of *NF1* somatic event observed in UESL is consistent with the non-NF1-associated status of this tumor.

Radical resection remains a key element in the successful treatment of UESL. Overall survival in patients with complete tumor resection is good (above 70%). In cases where the tumor cannot be removed due to multifocal lesions or the adherence of vital structures, the survival rates can be improved by the neoadjuvant chemotherapy or the patient can undergo liver transplantation (4, 24, 25). In the current clinical case, the patient was refractory to standard systemic chemotherapy without tumor response, whereas complete tumor resection with a liver transplant could not be performed due to periportal infiltration

Transarterial chemoembolization and chemoperfusion (notably the regional hepatic hemoperfusion) refer to infusions of chemotherapy drugs into local circulation of a particular organ. The procedure has been perfectly adapted for malignant tumors of the liver, notably hepatocellular carcinoma in adults, but also in pediatric patients. The method affords significant regression of hypervascular lesions, including those in chemoresistant and unresectable tumors (26). The combination of embolic intervention with chemotherapy allows precise delivery of the drug to the tumor while reducing systemic side effects and extending the actual duration of the drug action within the tumor. The biologically active chemoembolic formulations have been shown to persist in tumor tissues for up to 3 weeks. In addition, embolization of the nourishing vessels facilitates ischemic necrosis of the tumor, promoting not only its reduction in size, but also devascularization, which helps to reduce the blood loss during subsequent surgical resection (27, 28). In our patient, we encountered a robust response to TACE; the fatal outcome was associated with the synchronous development of a primary metastatic aggressive malignancy, which excluded effective surgical intervention for UESL.

The pathogenetic link between NF1 and UESL, firstly shown by us in this case report, remains disputable and requires deeper clinical insights, larger cohorts, and more advanced molecular profiling. The early-stage detection of MPNST and other tumors in NF1 remains the central task of the prevention schedules in patients with NF1, aimed at improving their quality of life and, ultimately, survival. The competitive synchronous development of highly aggressive soft tissue sarcoma dramatically interfered with the prognosis for UESL, which consistently responded to TACE with chemoperfusion. Based on this experience, TACE may be considered as a first-line treatment option for unresectable UESL and probably other solid neoplasms in pediatric patients with comorbidities and contraindications for systemic preoperative chemotherapy.

## Conclusion

We firstly report UESL in a patient with NF1, complicated by a synchronous tumor process. The clinical data demonstrate that TACE may represent an effective treatment option in pediatric patients with unresectable UESL. The prognosis was critically undermined by the second tumor (MPNST) of aggressive metastatic character. The key factor influencing the prognosis for patients with NF1 is adherence to clinical recommendations directed at early detection of neoplasms associated with this syndrome.

## Data availability statement

The original contributions presented in the study are included in the article/supplementary material. Further inquiries can be directed to the corresponding author.

## Ethics statement

The studies involving human participants were reviewed and approved by Local Ethics Committee of D. Rogachev National Medical Research Center of Pediatric Hematology, Oncology and Immunology. Written informed consent to participate in this study was provided by the participants’ legal guardian/next of kin. Written informed consent was obtained from the individual(s), and minor(s)’ legal guardian/next of kin, for the publication of any potentially identifiable images or data included in this article.

## Author contributions

KS, DL and AD: Conceptualized and drafted the initial manuscript. LY, DK, RA, YM, NU, AK and GN: Paper compilation and research. DA: Performed abdominal surgery and helped to draft the manuscript. AP and OM: Performed endovascular occlusion and helped to draft the manuscript. All authors contributed to the article and approved the submitted version.

## Funding

Molecular-genetic study was funded by the Foundation for support and development in the field of pediatric hematology, oncology and immunology “Science for Children”.

## Conflict of interest

The authors declare that the research was conducted in the absence of any commercial or financial relationships that could be construed as a potential conflict of interest.

## Publisher’s note

All claims expressed in this article are solely those of the authors and do not necessarily represent those of their affiliated organizations, or those of the publisher, the editors and the reviewers. Any product that may be evaluated in this article, or claim that may be made by its manufacturer, is not guaranteed or endorsed by the publisher.
